# Morphological and Molecular Diagnosis of Anisakid Nematode Larvae from Cutlassfish (*Trichiurus lepturus*) off the Coast of Rio de Janeiro, Brazil

**DOI:** 10.1371/journal.pone.0040447

**Published:** 2012-07-09

**Authors:** Juliana Novo Borges, Luiz Felipe Gullo Cunha, Helena Lúcia Carneiro Santos, Cassiano Monteiro-Neto, Cláudia Portes Santos

**Affiliations:** 1 Laboratório de Avaliação e Promoção e Saúde Ambiental, Instituto Oswaldo Cruz, Rio de Janeiro, Brasil; 2 Laboratório de Biologia do Nécton e Ecologia Pesqueira, Biologia Marinha, Universidade Federal Fluminense, Rio de Janeiro, Brasil; Institut national de la santé et de la recherche médicale - Institut Cochin, France

## Abstract

Anisakid nematode larvae from *Trichiurus lepturus* off coast of Rio de Janeiro were studied using light, laser confocal and scanning electron microscopy, in addition to a molecular approach. Mitochondrial cytochrome c-oxidase subunit 2 (mtDNA *cox*-2), partial 28S (LSU) and internal transcribed spacers (ITS-1, 5.8S, ITS-2) of ribosomal DNA were amplified using the polymerase chain reaction and sequenced to evaluate the phylogenetic relationships between the nematode taxa. The morphological and genetic profiles confirmed that, of the 1,030 larvae collected from the 64 fish examined, 398 were analysed, of which 361 were *Hysterothylacium* sp. and 37 were *Anisakis typica*. Larvae of *Hysterothylacium* sp. were not identified to the species level due to the absence of similar sequences for adult parasites; however, the ITS sequence clustered in the phylogenetic tree with sequences of *H. deardorffoverstreetorum,* whereas an mtDNA *cox*-2 and LSU concatenated phylogenetic analysis demonstrated the presence of two clades, both of them under the same name as the larval *H. deardorffoverstreetorum*. Data on the occurrence of parasites during the winter and summer months were compared using the t-test. The greatest prevalence and intensity of infection were recorded for larval *Hysterothylacium,* with a prevalence of 51.56% and an intensity of up to 55 parasites per fish. The larval *Anisakis* exhibit a higher abundance and intensity of infection in the winter months, and those of *Hysterothylacium* during the summer. However, the t-test indicated no significant differences between the abundance and intensity of infection recorded during the months of collection for either of these larval nematodes. All sequences generated in this study were deposited in GenBank.

## Introduction

Anisakid nematodes are parasites with an indirect life cycle, which utilizes hosts at different trophic levels in the food web. Aquatic vertebrates, such as piscivorous fishes, mammals and birds, are definitive hosts and aquatic invertebrates and fishes act as intermediate or paratenic hosts [1], [2]. The Anisakidae Skrjabin & Karokhin, 1945 is a major family within the Ascaridoidea Railliet & Henry, 1915, with species of *Anisakis* Dujardin, 1845, *Contracaecum* Railliet & Henry, 1912, *Pseudoterranova* Mozgovoi, 1950 and *Hysterothylacium* Ward & Magath, 1917 among the most reported as larvae in fishes [2], [3].

Anisakid larva are usually very difficult to identify to species using morphology due to the lack of differential characters, but when adults are already described and genetically characterized, then such larva can be assigned to a species based on molecules [1], [4]. The accurate identification of anisakid species is essential, because there are important pathogens within the group that can cause problems for human and animal health [2], [5], [6], [7]. Molecular tools are therefore valuable for linking anisakid larva to known adults as well as for systematic, evolutionary and ecological studies of these parasites [1], [4], [5], [8], [9].

The cutlassfish *Trichiurus lepturus* L. (Trichiuridae) has a wide distribution, occurring throughout tropical and temperate waters of the world. Previous parasitological surveys on specimens from off the coast of Rio de Janeiro listed the occurrence of anisakid larva identified only to generic level by means of light microscopy [10], [11]. In this study, the nematode parasites of *T. lepturus* from the same region are re-evaluated using light, laser confocal and scanning electron microscopy, and also by the determination of nucleotide sequences from the internal transcribed spacers of ribosomal DNA (ITS-1, 5.8S, ITS-2), the partial 28S (LSU) and mitochondrial cytochrome c-oxidase subunit 2 (mtDNA *cox*-2).

## Results

A total of 1,030 nematode larva were collected from 64 fishes; 398 were analyzed for morphological data and 72 were used for genetic studies. The larvae identified by morphological and molecular approaches as *Anisakis typica* and *Hysterothylacium* sp. are characterized below.

### 

#### *Anisakis typica* third-stage larva

**Figure 1:**
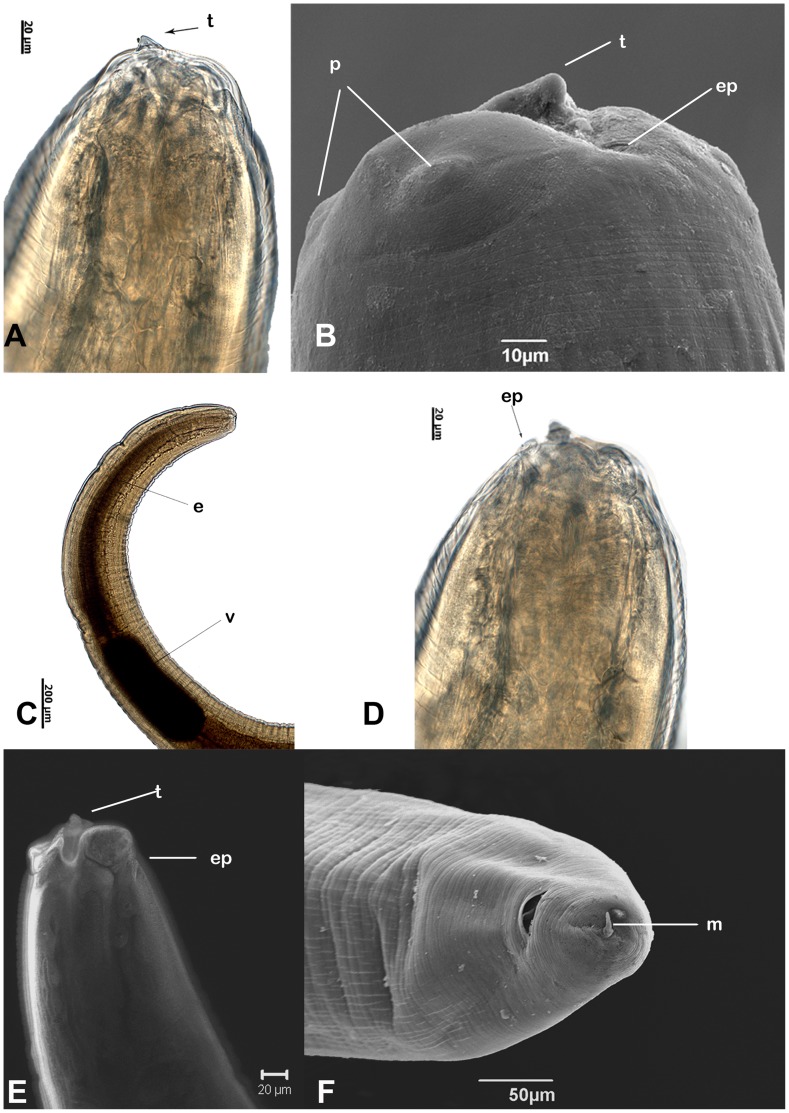
A–F: *Anisakis typica* larvae: light, CLSM and SEM microscopy. A- Aanterior end with boring tooth; B- SEM of lips with papilla, boring tooth and excretory pore; C- Esophagus and ventriculus; D- Position of excretory pore; E- CLSM reconstruction with detail boring tooth and excretory pore; F- SEM of tail with mucron terminal. Abbreviations: e - esophagus; ep - excretory pore; t - tooth; p - papilla; v - ventriculus; m - mucron.

Thirty seven specimens were collected from the body cavity and mesentery of *T. lepturus;* their measurements are presented in Table 1. They had the following characteristics: cuticle smooth; lips poorly developed; ventrolateral lips with single and double papilla, dorsal lip with two double papillae; boring tooth present between ventral lips; intestinal caecum absent; ventriculus elongate (Figures 1A–1C). Excretory pore present at the base of ventrolateral lips (Figures 1B, 1D–1E); tail short, round, with mucron (Figure 1F).

**Table 1:** Present measurements of *Anisakis typica* and *Hysterothylacium* sp.

	*Anisakis typica* L3 (n = 12)	*Hysterothylacium* sp. L3 (n = 28)	*Hysterothylacium* sp. L4 (n = 13)
Body length	19.31 (15.34–22.43)	7.84 (3.42–14.8)	9.17 (6.55–11.55)
Body width	0.45 (0.6–0.35)	0.24 (0.13–0.4)	0.27 (0.14–0.35)
Esophagus	1.46 (1.81–1.1)	0.64 (0.41–0.87)	0.77 (0.6–0.98)
Ventriculus	0.61 (0.76–0.5)	0.07 (0.04–0.1)	0.09 (0.06–0.1)
Intestinal caecum	Absent	0.16 (0.1–0.46)	0.28 (0.15–0.4)
Ventricular appendix	Absent	0.59 (0.31–0.84)	0.67 (0.42–0.94)
Tail	0.12 (0.2–0.08)	0.16 (0.11–0.22)	0.16 (0.11–0.25)
Esophagus/ventriculus	1∶0.30–0.54	1∶0.07–0.20	1∶0.07–0.14
Esophagus/caecum	–	1∶0.14–0.34	1∶0.20–0.57
Esophagus/ventricular appendix	–	1∶0.60–1.33	1∶ 0.55–1.00

**Figure 2:**
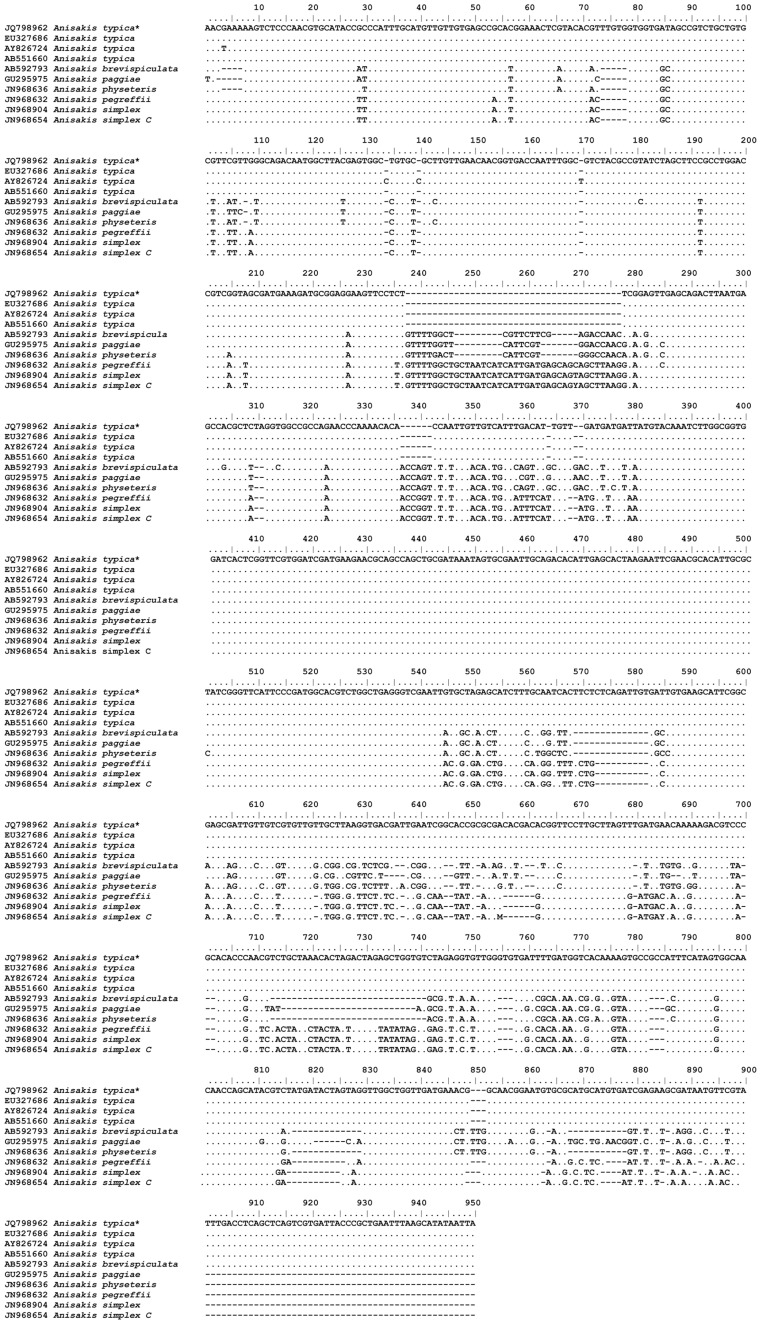
Alignment of ITS-1 and ITS-2 sequences representing *Anisakis* spp. Dots indicate identity with the first sequence, dashes are inferred insertion-deletion events and * represents our sample.

**Figure 3:**
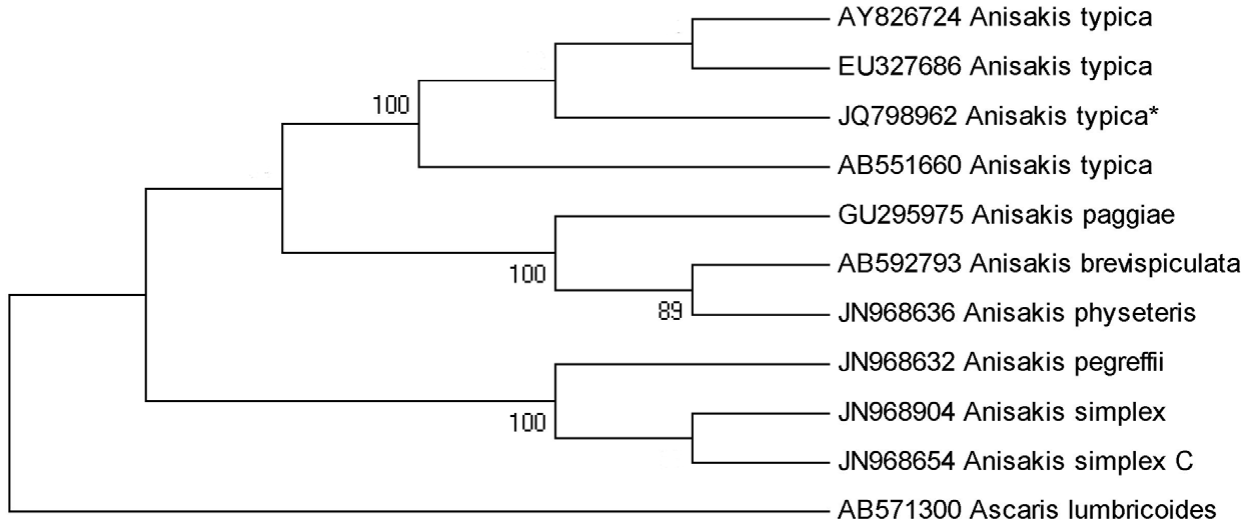
Maximum likelihood reconstruction between sequences of *Anisakis typica* obtained in this study (*) and sequences of *Anisakis* species from the GenBank, with the tree inferred from the ITS data set. The numbers on the tree branches represent the percentage of bootstrap resampling. *Ascaris lumbricoides* was used as an out group.

**Figure 4:**
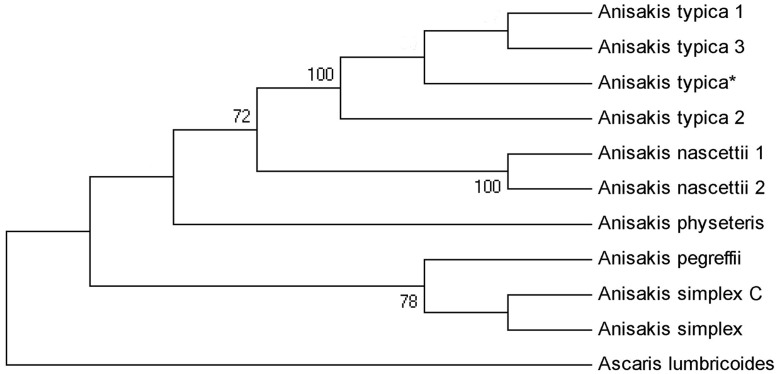
Maximum likelihood reconstruction between sequences of *Anisakis typica* obtained in this study (*) and sequences of *Anisakis* species from the GenBank, with the tree inferred from mtDNA *cox*-2 and LSU data sets. The numbers on the tree branches represent the percentage of bootstrap resampling. *Ascaris lumbricoides* was used as an out group.

Genetic characterization of 22 larva enabled the species determination, with 13 being diagnosed by specific PCR as *Anisakis typica* (Diesing, 1860); 9 were submitted to PCR for family for each genetic region (ITS, mtDNA *cox*-2 and LSU) with subsequent sequencing reactions. Considering only those sequences of suitable quality for the genetic characterization, five sequences were obtained for the ITS region, one for mtDNA *cox*-2 region and three for the LSU region (accession n. JQ798962, JQ798968 and JQ798967, respectively). The alignment of the sequences from this study with sequences from GenBank resulted in 100% of similarity for sequences of the ITS region (Figure 2) and 99% for sequences of the mtDNA *cox*-2 region. There were no previous sequences for the LSU region of *Anisakis typica* deposited on GenBank for comparison. Phylogenetic analysis for *A. typica* demonstrated a clear separation between different species of *Anisakis* with strong statistical support (Figures 3 and 4). This is the first identification of *A. typica* in *T. lepturus* in Brazilian waters, with the new LSU sequence being deposited in the GenBank.

#### *Hysterothylacium* sp. third and fourth-stage larvae

**Figure 5:**
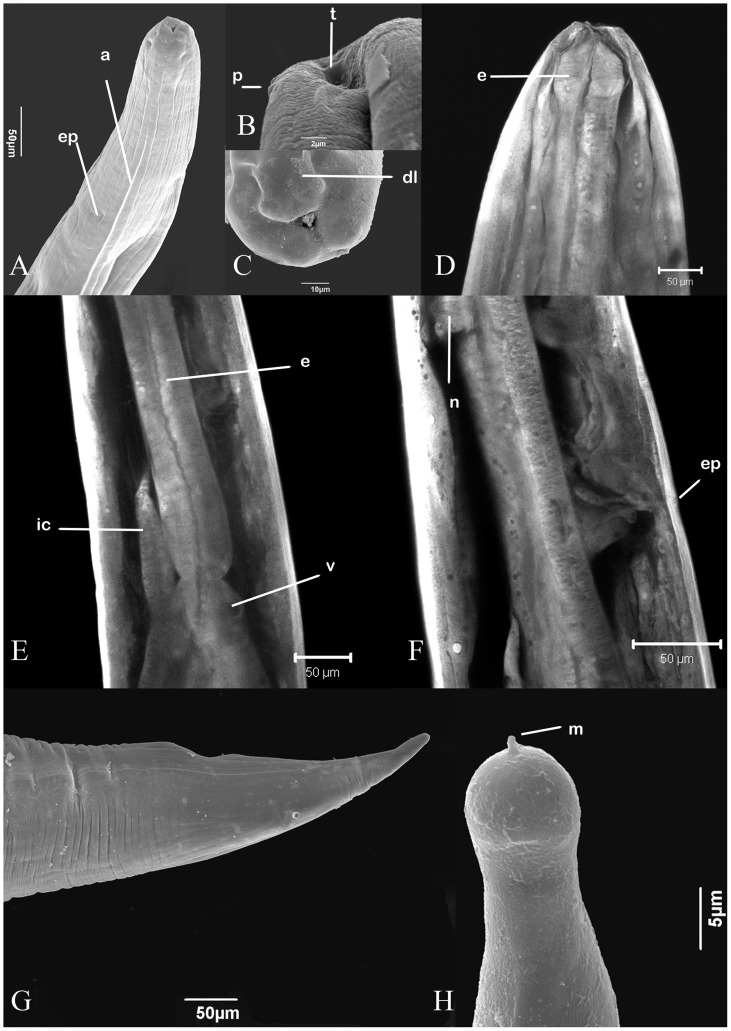
A–H: *Hysterothylacium* sp. larvae: SEM and CLSM microscopy. A- SEM of anterior end with alae and excretory pore; B- Detail of L3 lips with inconspicuous boring tooth and papillae; C- Detail of lips of L4 with dorsal lip showing double papilla; D- CLSM of esophagus; E- CLSM reconstruction with ventriculus, intestinal caecum and esophagus; F: CLSM reconstruction with nerve ring and excretory pore; G- SEM of tail; H- SEM micrograph with a detail of the digitiform tip with terminal mucron. Abbreviations: a - alae; ep - excretory pore; p – papilla; t - tooth; dl - dorsal lip; e - esophagus; ic - intestinal caecum; v - ventriculus; n - nervous ring and m - mucron.

Three hundred sixty one specimens were collected from the body cavity and mesentery of the cutlassfish. Measurements were taken from 28 L3 and 13 L4 individuals (Table 1). They had the following characteristics: small worms, with smooth cuticle and distinct lateral alae along each side of body between level just posterior to lips and pre-cloacal region (Figure 5A). L3 with anterior region rounded and lacking defined lips; inconspicuous boring tooth present (Figure 5B); L4 with developing lips and lacking boring tooth (Figure 5C); esophagus claviform; ventriculus small and rounded (Figures 5D–5F); intestinal caecum smaller than ventricular appendix; excretory pore inconspicuous, located between nerve ring and anterior extremity of intestinal caecum, clearly visible in SEM and CLSM images (Figures 5A, 5F); tail long, digitiform, with terminal mucron (Figures 5G–5H).

**Figure 6:**
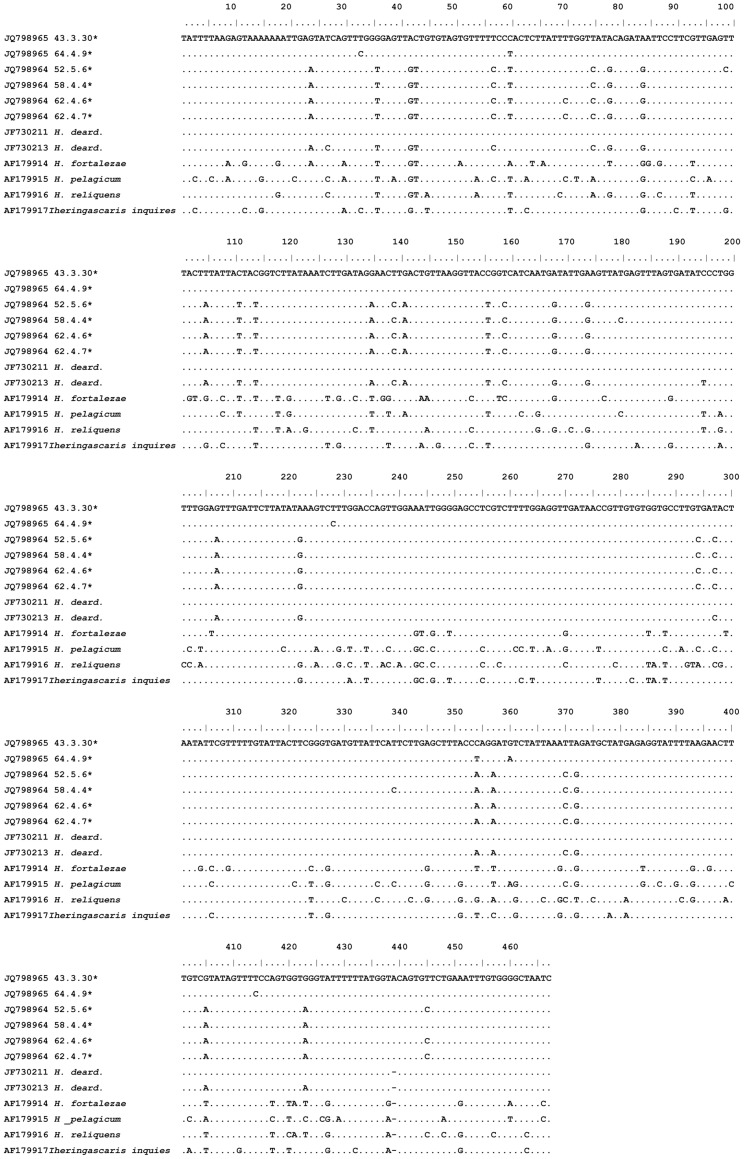
Alignment of mtDNA *cox*-2 sequences representing *Hysterothylacium* and *Iheringascaris* taxa. Dots indicate identity with the first sequence, dashes are inferred insertion-deletion events and * represents our samples.

**Figure 7:**
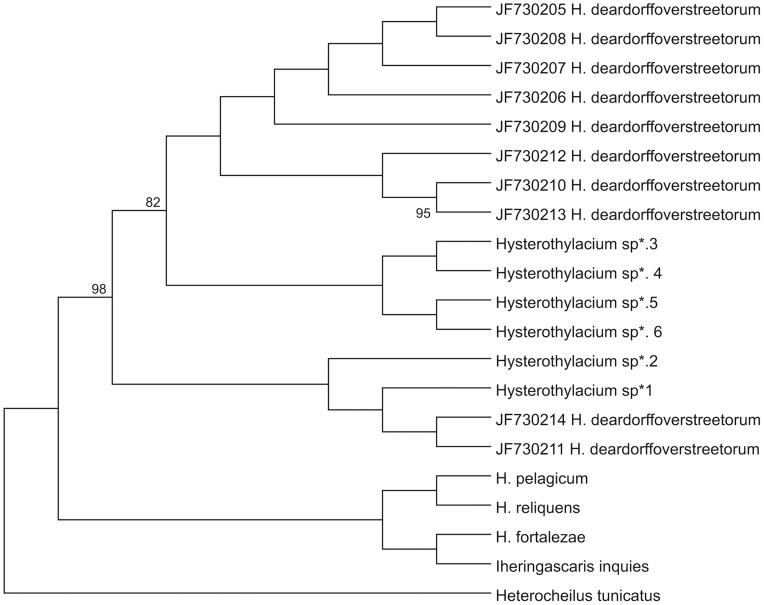
Maximum likelihood reconstruction between sequences of *Hysterothylacium* obtained in this study (*) and sequences of *Hysterothylacium* and *Iheringascaris* spp. from the GenBank, with the tree inferred from mtDNA *cox*-2 and LSU data sets. The numbers on the tree branches represent the percentage of bootstrap resampling. *Heterocheilus tunicatus* was used as an out group.

**Figure 8:**
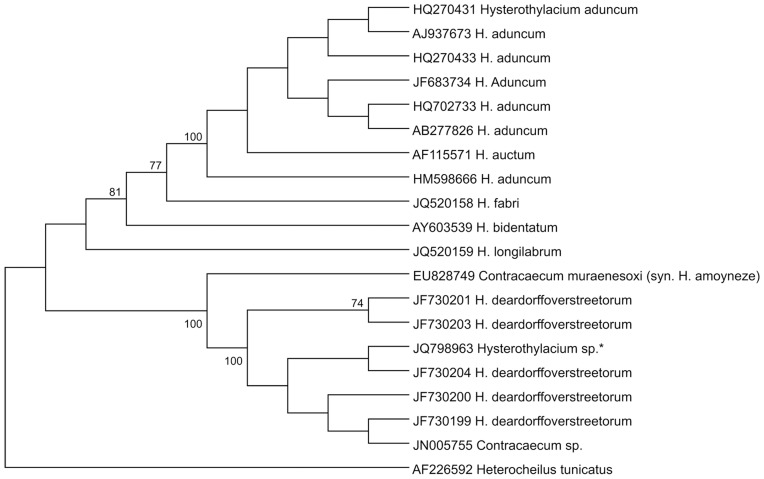
Maximum likelihood reconstruction between sequences of *Hysterothylacium* sp. larvae obtained in this study (*) and sequences of other anisakid species from the GenBank inferred from the ITS dataset. The numbers on the tree branches represent the percentage of bootstrap resampling. *Heterocheilus tunicatus* was used as an out group.

Genetic characterization of 41 larva with PCR for family for each genetic region (ITS, mtDNA *cox*-2 and LSU) and DNA sequencing was carried out, and 17 good quality sequences were obtained for the ITS region, 30 for the mtDNA *cox*-2 region and 19 for the LSU region (accession nos. JQ798963, JQ798964, JQ798965 and JQ798966). The alignment of these sequences, with reference sequences (Table 2), resulted in a 100% of similarity for the closest sequence of the ITS region (JF730200), 99% for the closest sequence of the mtDNA *cox*-2 region (JF730213 and JF730211) (Figure 6) and 96% for the closest sequence of the LSU region (AY821772). The mtDNA *cox-*2 sequences obtained presented a maximum pairwise distance of 8% when compared with reference sequences (Table 2). The pairwise distance among our mtDNA *cox*-2 sequences had a maximum distance of 9%. An mtDNA *cox*-2 and LSU concatenated phylogenetic analysis (Figure 7) of sequences of *Hysterothylacium* specimens studied in the present paper demonstrated the presence of two clades, both of them including sequences under the name of *H. deardorffoverstreetorum* retrievable from GenBank (Figure 7). The ITS phylogenetic tree was performed with a single sequence of *Hysterothylacium*; this sequence obtained in the present paper clustered in a single clade, well supported, including all the sequences of *H. deardorffoverstreetorum* deposited in GenBank (Figure 8).

**Table 2:** List of species from the *Genbank* used for comparison in phylogenetic analysis and alignments.

Genetic region	Species	GenBank acession number	Reference
ITS	*Contracaecum sp.*	JN005755	Unpublished data
	*Contracaecum muraenesoxi*	EU828749	Fang *et al*. 2009 Exp. Parasitol.
	*Hysterothylacium aduncum*	HQ270433	Amor *et al*. 2011 Parasitol. Res.
	*Hysterothylacium aduncum*	HQ270431	Amor *et al*. 2011 Parasitol. Res.
	*Hysterothylacium aduncum*	JF683734	Unpublished data
	*Hysterothylacium aduncum*	HQ702733	Unpublished data
	*Hysterothylacium aduncum*	AJ937673	Zhu *et al*. 2007 Parasitol. Res.
	*Hysterothylacium aduncum*	HM598666	Unpublished data
	*Hysterothylacium aduncum*	AB277826	Umehara *et al*. Parasitol. Int.
	*Hysterothylacium auctum*	AF115571	Szostakowska *et al*. 2001 Acta Parasitol.
	*Hysterothylacium bidentatum*	AY603539	Unpublished data
	*Hysterothylacium deardorffoverstreetorum*	JF730200	Knoff *et al*. 2012 Mem. Inst. Oswaldo Cruz
	*H. deardorffoverstreetorum*	JF730201	Knoff *et al*. 2012 Mem. Inst. Oswaldo Cruz
	*H. deardorffoverstreetorum*	JF730203	Knoff *et al*. 2012 Mem. Inst. Oswaldo Cruz
	*H. deardorffoverstreetorum*	JF730204	Knoff *et al*. 2012 Mem. Inst. Oswaldo Cruz
	*H. deardorffoverstreetorum*	JF730199	Knoff *et al*. 2012 Mem. Inst. Oswaldo Cruz
	*Hysterothylacium fabri*	JQ520158	Li *et al*. 2012 Parasitol. Res.
	*Hysterothylacium longilabrum*	JQ520159	Li *et al*. 2012 Parasitol. Res.
	*Anisakis brevispiculata*	AB592793	Murata *et al*. 2011 Parasitol. Int.
	*Anisakis paggiae*	GU295975	Klimpel *et al*. 2011 Polar Biol.
	*Anisakis physeteris*	JN968636	Kuhn *et al*. 2011 Plos One
	*Anisakis pegreffii*	JN968632	Kuhn *et al*. 2011 Plos One
	*Anisakis simplex*	JN968904	Kuhn *et al*. 2011 Plos One
	*Anisakis simplex C*	JN968654	Kuhn *et al*. 2011 Plos One
	*Anisakis typica*	AY826724	Unpublished data
	*Anisakis typica*	AB551660	Umehara *et al*. 2010 Int. J. Food microbiol.
	*Anisakis typica*	EU327686	Iñiguez *et al*. 2009 Vet. Parasitol.
	*Ascaris lumbricoides*	AB571300	Arizono *et al*. 2010 Jpn. J. Infect. Dis.
	*Heterocheilus tunicatus*	AF226592	Nadler *et al*. 2000 Parasitol.
LSU	*Hysterothylacium pelagicum*	AF226590	Nadler *et al*. 2000 Parasitology
	*Hysterothylacium fortalezae*	U94760	Nadler & Hudspeth 1998 Mol. Phylogenet.
	*Hysterothylacium reliquens*	U94762	Nadler & Hudspeth 1998 Mol. Phylogenet.
	*Iheringascaris inquies*	U94763	Nadler & Hudspeth 1998 Mol. Phylogenet.
	*Anisakis simplex C*	AY821754	Nadler *et al*. 2005 J. Parasitol.
	*Heterocheilus tunicatus*	AF226592	Nadler *et al*. 2000 Parasitol.
	*Asacaris lumbricoides*	AF182298	Nadler & Hudspeth 2000 J. Parasitol.
mtDNA cox2	*Hysterothylacium fortalezae*	AF179914	Nadler & Hudspeth 1998 Mol. Phylogenet.
	*Hysterothylacium deardorffoverstreetorum*	JF730211	Knoff *et al*. 2012 Mem. Inst. Oswaldo Cruz
	*H. deardorffoverstreetorum*	JF730213	Knoff *et al*. 2012 Mem. Inst. Oswaldo Cruz
	*H. deardorffoverstreetorum*	JF730205	Knoff *et al*. 2012 Mem. Inst. Oswaldo Cruz
	*H. deardorffoverstreetorum*	JF730208	Knoff *et al*. 2012 Mem. Inst. Oswaldo Cruz
	*H. deardorffoverstreetorum*	JF730207	Knoff *et al*. 2012 Mem. Inst. Oswaldo Cruz
	*H. deardorffoverstreetorum*	JF730206	Knoff *et al*. 2012 Mem. Inst. Oswaldo Cruz
	*H. deardorffoverstreetorum*	JF730209	Knoff *et al*. 2012 Mem. Inst. Oswaldo Cruz
	*H. deardorffoverstreetorum*	JF730212	Knoff *et al*. 2012 Mem. Inst. Oswaldo Cruz
	*H. deardorffoverstreetorum*	JF730210	Knoff *et al*. 2012 Mem. Inst. Oswaldo Cruz
	*H. deardorffoverstreetorum*	JF730214	Knoff *et al*. 2012 Mem. Inst. Oswaldo Cruz
	*Hysterothylacium pelagicum*	AF179915	Nadler & Hudspeth 1998 Mol. Phylogenet.
	*Hysterothylacium reliquens*	AF179916	Nadler & Hudspeth 1998 Mol. Phylogenet.
	*Iherigascaris inquies*	AF179917	Nadler & Hudspeth 1998 Mol. Phylogenet.
	*Anisakis typica 1*	AB517571	Suzuki *et al*. 2009 Int. J. Food Microbiol.
	*Anisakis typica 2*	AB517572	Suzuki *et al*. 2009 Int. J. Food Microbiol.
	*Anisakis typica 3*	DQ116427	Valentini *et al*. 2006 J. Parasitol.
	*Anisakis nascettii 1*	GQ118169	Mattiucci *et al*. 2009 Syst. Parasitol.
	*Anisakis nascettii 2*	GQ118171	Mattiucci *et al*. 2009 Syst. Parasitol.
	*Anisakis simplex*	HM488999	Setyobudi *et al*. 2011 Parasitol. Res.
	*Anisakis pegreffii*	JF423263	Baldwin *et al*. 2011 J. Parasitol.
	*Anisakis physeteris*	AB592801	Murata *et al*. 2011 Parasitol. Int.
	*Heterocheilus tunicatus*	AF179913	Nadler *et al*. 2000 J. Parasitol.
	*Ascaris lumbricoides*	AF179907	Nadler & Hudspeth 2000 J. Parasitol.

### Ecological Data

**Figure 9:**
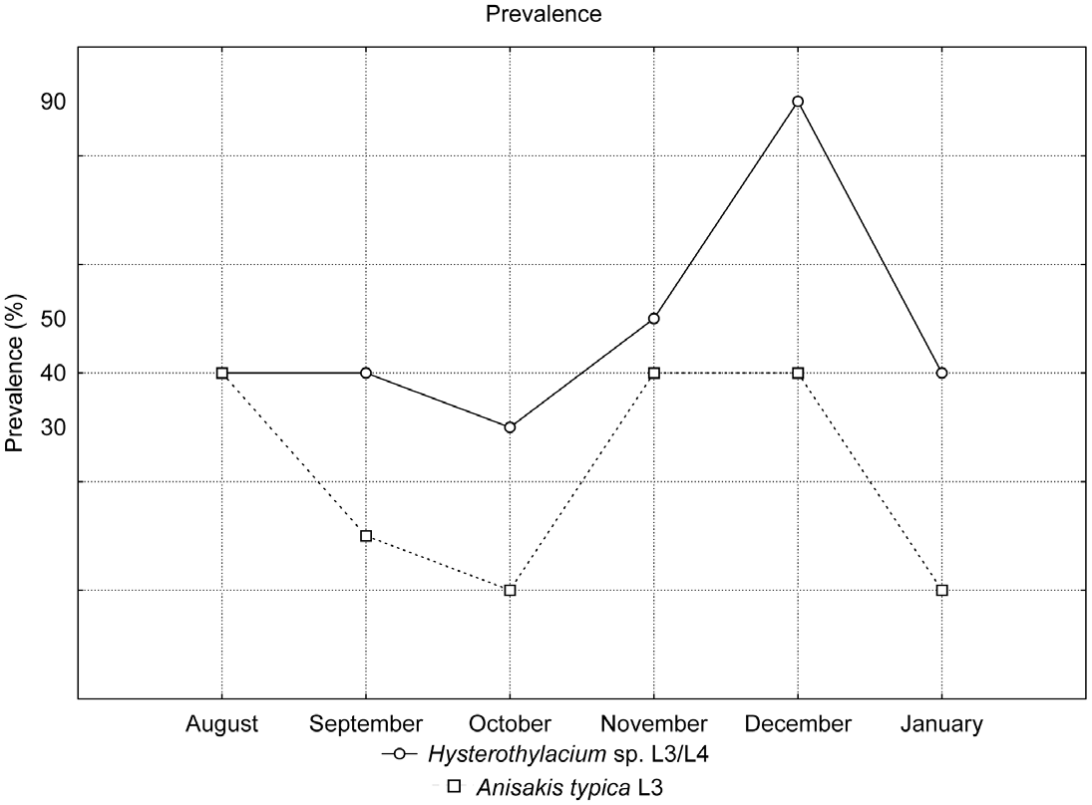
Ecological data of *Anisakis typica* and *Hysterothylacium* sp.: prevalence expressed as a percentage.

**Figure 10:**
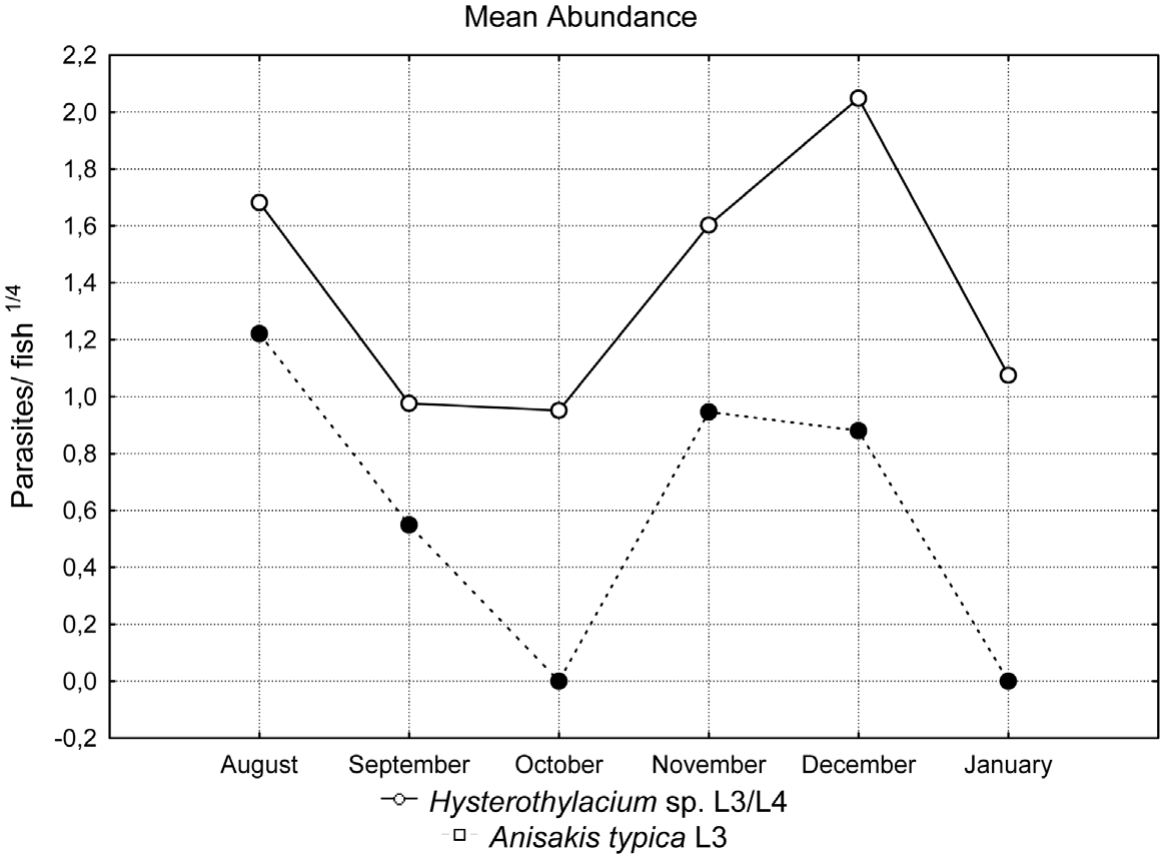
Ecological data of *Anisakis typica* and *Hysterothylacium* sp.: mean abundance (no. of parasites/fish) transformed using the fourth root.

**Figure 11:**
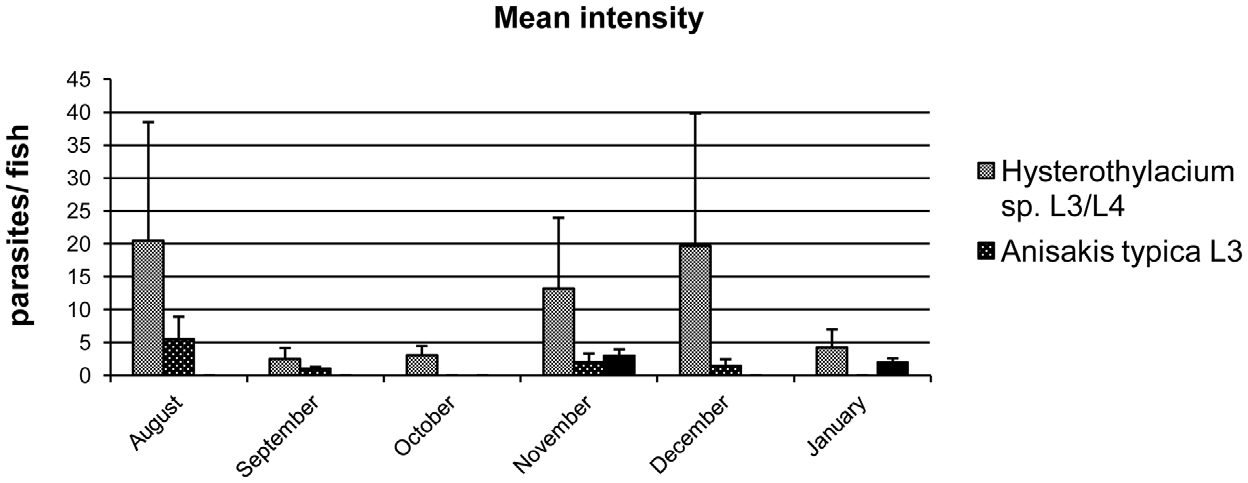
Ecological data of *Anisakis typica* and *Hysterothylacium* sp.: mean intensity (no. of parasites/parasitized fish); the bars represent the standard deviation.

The prevalence of *Anisakis typica* was 20.31% and the intensity varied from 1 to 10 specimens per fish. *Hysterothylacium* presented a prevalence of 51.56% and intensity of 1 to 55 per fish. The highest prevalences were found during November and December (Figure 9). Larvae of *Hysterothylacium* sp. were the most abundant, with mean intensities between 2.5 and 20.5 (Figures 10 and 11). The t-test applied to verify the existence of variation between prevalence, intensity and abundance during winter (August) and summer (December and January) was not significant.

## Discussion

The larvae of *Hysterothylacium* sp. are difficult to identify and their similarity with related genera has resulted in taxonomic confusion, with species of *Hysterothylacium* being identified as *Contracaecum* or *Iheringascaris* [12], [13], [14]. The position of the excretory pore, which has been reported as inconspicuous, is the main difference between larvae of *Hysterothylacium* (positioned at nerve ring level) and *Contracaecum* (situated at the base of lips). As mentioned above, the confocal microscopy and SEM were essential to ascertain its location. Likewise, species of *Iheringascaris* are separated from *Hysterothylacium* based only in the pattern of annulations of the cuticle [12]. In the present study, the SEM micrographs showed the cuticle to lack annulations, as described for *Hysterothylacium* spp., although the phylogenetic analysis showed a close relationship with *Iheringascaris*. In the future, it is possible that species of *Iheringascaris* may be allocated within *Hysterothylacium* [15].

In this study, larvae of *Hysterothylacium* are reported at a high prevalence (51.56%), with an intensity of infection of up to 55 parasites per fish, but could not be identified to species level due to the absence of related adult sequences in the GenBank. Consequently, a specific identification could not be assigned.

Previous genetic analysis of *Anisakis simplex* and *Hysterothylacium aduncum* from *T. lepturus* in Taiwanese waters [16] were described but not formally deposited in the GenBank. However, a comparison with these data showed these species are genetically distinct from the nucleotide sequences obtained in this study.

The similarity among our *Hysterothylacium* sequences for ITS and LSU regions was 100%; on the contrary, our mtDNA *cox*-2 sequences exhibited a high genetic heterogeneity. The presence of polymorphism in the mtDNA *cox*-2 region has likewise been reported before for other species of nematodes [17]. The K2P distances calculated among the sequences available in GenBank under the name of *H. deardorffovertsreetorum* and the *Hysterothylacium* sequenced here, showed a genetic differentiation ranging from K2P = 0.005 to K2P = 0.08. The present study indicates that the *Hysterothylacium* larvae analyzed were likely to correspond to the larva described as *H. deardorffoverstreetorum*; however, the marked genetic differentiation so far detected at the mtDNA *cox*-2 level seems to suggest a possible genetic heterogeneity. This needs to be further investigated by future genetic analysis, likely using other nuclear markers. Indeed, while a comparison with one of the sequences of *H. deadorffoverstreetorum* (accession no. JF730200) resulted in a 100% of similarity for the ITS region, the mtDNA *cox*-2 sequences deposited, under the same name, had, at the intraspecific level, a genetic differentiation value with K2P distances ranging from 0.002 to 0.077. This value has been also found among the sequences of *Hysterothylacium* analysed here at the same gene (K2P up to 0.092). Interestingly, the value of K2P = 0.07 is generally observed at the mitochondrial level between sibling or cryptic species of other anisakid nematodes [1], [18]. Therefore, future genetic studies will no doubt clarify the genetic heterogeneity indicated here using nuclear markers.

On the other hand, the morphology of larvae, especially of sibling species, appears to be often overlapping and not fully diagnosed when not accompanied by the genetic methodological approaches.

There are about 60 species of *Hysterothylacium* which have been formally described based on the morphological features of the adult worm [19], [20], [21], [22]. However, so far, scanty data are available for their molecular analysis. *Hysterothylacium* sequences determined in this work were not similar to those deposited in the GenBank based on adult characterization. The question remains: could it be a new species, as indicated by the phylogenetic analysis, or a known species based on the morphological features of an adult worm which has not yet been characterized by molecular means? Species descriptions should contain data from as many sources as possible, including morphological infomation from adult worms, molecular data and phylogenetic analyses, which can be used not only as tools for identifying an isolate specimen but also for understanding its biology and taxonomy.

*Hysterothylacium* sp. type MB larvae *sensu* Deardorff and Overstreet [12] were reported from *T. lepturus* in the Sea of Oman [23], but the authors refrained from naming it. Similarly, unknown anisakid larvae have been reported from fishes using a PCR-based approach as evidence for new species, but the new form was not formally described as adults were not available for morphological characterization and molecular comparison [24]. However, *Hysterothylacium deardorfoverstreetorum* has recently been proposed based only on morphological features of the larva and a comparison with sequences of the genus deposited in the GenBank, despite their small number [25]. It is possible that, in future when sequences of adults of all or most of the 60 nominal species of *Hysterothylacium* are deposited in the GenBank, this species will likely sink into synonymy, reinforcing the idea that molecular data should be accompanied by strong morphological evidence based on adult nematodes.

The genotyping of more species will enable GenBank to become a robust tool for identification and phylogenetic analyses. However, at present, the number of sequences of *Hysterothylacium* deposited in this database represents less than 15% of the valid species. This limitation compromises any phylogenetic results when the objective is to identify a species. For this, it is necessary to characterize a larger number of valid species based on genotypic information and morphological analyses of adult worms in order to enable the genetic identification of *Hysterothylacium* larvae.

An ITS sequence of the larvae of *Contracaecum* sp. found in *Pagellus bogaraveo* in Portuguese waters (accession no. JN005755) also presented 99% similarity with *Hysterothylacium* sequences from this study. Unfortunately, a formal publication with morphological characterization was not available for comparison.

Within the GenBank, the ITS sequence (accession no. EU828749) identified as *Contracaecum muraenesoxi* appeared to be very closely related to the sequences determined in this study. Nevertheless, this species was recently synonymized with *Hysterothylacium amoyneze* [21], explaining its phylogenetic position within the *Hysterothylacium* and proximity to our sequence. This highlights the fact that taxonomic changes of taxonomic names need, somehow, to be included in the GenBank in order to avoid phylogenetic misinterpretation. Similarly, the phylogenetic analysis showed an LSU sequence of *Raphidascaris acus* (accession no. AY821772) to be closely related to *Hysterothylacium* sp. from this study, which suggests that the morphological identification of that voucher of *R. acus* should be revised [15].

In this study, *Anisakis typica* was identified by molecular data, and our phylogenetic analysis for *Anisakis* species also indicated three distinct groups of species, agreeing with data from the literature [1].

The prevalence of *Anisakis* and *Hysterothylacium* larvae in this study were similar to those previously described in the cutlassfish off the coast of Rio de Janeiro [11]. Significant differences in prevalence were not observed between the winter and summer periods, although a moderate increase in prevalence and abundance was observed at the beginning of summer for *Hysterothylacium*. The prevalence of *Anisakis simplex* in fishes from Norway, for comparison, was most significant during spring, and the authors have suggested that a small variation in the occurrence of anisakids in tropical waters could be related to the low level of climatic variability typical for tropical weather [26]. The constant presence of definitive hosts along the Brazilian coast may also contribute to the presence of *Anisakis* and *Hysterothylacium* during both winter and summer, as observed in this study. *Hysterothylacium* adults have been reported off the Brazilian coast parasitizing the following definitive hosts: *Harengula clupeola*, *Scomberomorus cavalla*, *S. maculatus*, *Epinephelus guttatus* [27]. These definitive hosts have a preference for coastal habitats, which may be related to the prevalence and abundance of *Hysterothylacium* in *T. lepturus*.

Adults of *Anisakis typica* were described from the dolphins *Sotalia guianensis* and *Stenella longirostris* off the Brazilian coast. *S. guianensis* inhabits coastal waters, whereas *S. longirostris* prefers oceanic bays and island regions. *A. typica* larvae has been reported in *Thunnus thynnus* and *Auxis thazard* off Rio de Janeiro [28], [29], [30], [31], [32], indicating that the parasite is common in the area. During summer, there is an increase in whale-watching along the Rio de Janeiro coast, which is probably related to the seasonal upwelling in the region responsible for the addition of new elements to the food webs. At this time these food webs become more complex, thus promoting anisakid transmission [33], [34]. This may explain the increasing abundance of these parasites in the summer. Furthermore, the increase in prevalence of anisakids off the coast during spring and summer could be due to the spawning period of *T. lepturus*, whose foraging behaviour increases in order to build resources for reproduction [35].

This is the first identification of *A. typica* in *T. lepturus* in Brazilian waters with LSU, ITS and mtDNA *cox-*2 sequences for larvae of both of *A. typica* and *Hysterothylacium* sp. This integrated study has shown the great need for a linkage between the analysis of morphological features supplemented by molecular data in order to enable the accurate identification of anisakid larva and provide robust taxonomic data.

## Materials and Methods

A total of 64 fish were collected off Itaipu beach, Niterói, Rio de Janeiro (22°53′14′′S; 43°22′48′′O) from August 2010 to January 2011. Prevalence, abundance and mean intensity were calculated [36]. Data were transformed to attend the assumption of normality, and t- tests for independent samples were performed to verify differences between winter and summer months.

Nematodes were cut into three pieces and fixed in 70% ethanol. The anterior and posterior regions were cleared in glycerine and mounted as semi-permanent preparations on slides; the middle regions were used for molecular analyses. Specimens were examined using an Olympus CX3 microscope, and measurements were made with the aid of an ocular micrometer are given in micrometres as the mean, followed in parentheses by the range. High resolution confocal images were made using a confocal laser scanning microscope (Zeiss Axiovert 510, META). For scanning electron microscopical observations, some specimens were fixed for 24 hours at 4°C in a solution containing 2.5% glutaraldehyde and 4% paraformaldehyde in 0.1 M cacodylate buffer containing 3% sucrose at pH 7. The samples were washed in the same buffer and post-fixed overnight in 1% osmium tetroxide in 0.1 M cacodylate buffer at pH 7.2 in the dark. The specimens were dehydrated in an ethanol series, critical point dried with CO_2_, coated with 60 nm of gold and observed in a Jeol JSM 6390 SEM microscope.

The middle parts of parasites were prepared for total genomic DNA extraction using a ChargeSwitch gDNA Mini Tissue Kit (Invitrogen, Carlsbad, CA, EUA) according to the manufacturer’s instructions. To amplify gene fragments of anisakid nematodes, a set of primers were used: NC5/NC2 [37] for ITS fragments, 211F/210R [15] for mtDNA *cox-*2 gene fragments and 391/390 [5] for 28S rDNA gene fragments. The primer ITSF/ITSR was used to amplify the ITS region of *A. typica* [32]. All PCR reactions were performed in a volume of 50 µl with 20 mM of Tris-HCl at pH 8.4; 50 mM of KCl; 250 µM of each deoxynucleoside triphosphate (dNTPs) and 2 µl of genomic DNA. The concentrations of MgCl_2_, primers and Taq Gold DNA polymerase (Promega Hot Taq Go Start Madison, WII - USA) were different for each reaction: NC5/NC2 (1.5 µM of MgCl_2_, 0.5 µM of each oligonucleotide primer and 1 U of Taq); ITSF/ITSR (2.5 µM of MgCl_2_, 0.4 µM of each oligonucleotide primer and 1 U of Taq); 211F/210R (0.5 µM of forward and 0.4 µM of reverse oligonucleotides, 2.5 µM of MgCl_2_ and 1 U of Taq) and for 391/390 (0.4 µM of each oligonucleotide 3 µM of MgCl_2_ and 1.5 U of Taq). PCR was carried out using a Mastercycler Personal/Eppendorf thermal cycler (Epperdorf, Hamburg, Germany) and cycling parameters as previously described [5], [15], [32], [37].

PCR products were visualized with GELRED (Biotium Inc, Hayward, CA, USA) staining after electrophoresis on 1.5% agarose gels. All amplified PCR products generated were purified with Wizard SV gel and PCR clean up system kit (Promega) following the manufacturer’s instructions and sequenced in both directions using the same primer sets as in the respective PCR assay. DNA cycle-sequencing reactions were performed using BigDye v.3.1 chemistry (Applied Biosystems, Foster City, CA, USA). Sequencing reactions were performed in the ABI Prism 3100 sequence analyzer. Sequences were assembled, edited, in DNASTAR SeqMan (DNASTAR, Inc., Madison, WI), and aligned with Bioedit Sequence Alignment Editor (version 7.0.4.1; http://www.mbio.ncsu.edu/Bio Edit/bioedit.html). The edited sequences were compared for similarities with sequences from GenBank using BLAST 2.0 (“Basic Local Alignment Search Tool”) (Table 2) [38]. To examine the phylogenetic relationships, the nucleotide sequences were analyzed by CLUSTAL W algorithm of Bioedit Package [39], [40]. The sequences of the two mitochondrial genes (mtDNA *cox*-2 and LSU) were joined using the software Concatenator [41]. Phylogenetic trees were inferred by using the software MEGA 5.0 [42] utilizing the General Time Reversible model (GTR) for ITS sequences and Hasegawa-Kishino-Yano model (HKY) for mtDNA *cox*-2 and LSU. These models were selected using the program jModelTest [43]. Kimura Two Parameters (K2P) values were calculated by the software MEGA 5.0 [42], [44]. Maximum Likelihood method was used to construct trees [45] and were resampled by 100 bootstrap replicates to evaluate the reliability of the groups.
